# Dominant serotype distribution and antimicrobial resistance profile of *Shigella* spp. in Xinjiang, China

**DOI:** 10.1371/journal.pone.0195259

**Published:** 2018-04-03

**Authors:** Hongbo Liu, Binghua Zhu, Shaofu Qiu, Yidan Xia, Beibei Liang, Chaojie Yang, Nian Dong, Yongrui Li, Ying Xiang, Shan Wang, Jing Xie, Muti Mahe, Yansong Sun, Hongbin Song

**Affiliations:** 1 Academy of Military Medical Sciences, Academy of Military Sciences, Beijing, China; 2 Institute of Disease Control and Prevention, PLA, Beijing, China; 3 Center for Disease Control and Prevention of Xinjiang Uygur Autonomous Region, Urumqi, China; Beijing Institute of Microbiology and Epidemiology, CHINA

## Abstract

*Shigella* represents one of the major diarrhea-inducing pathogens threatening public health, but its prevalence and antimicrobial resistance profile in Xinjiang Uygur Autonomous region, China, remains unclear. We conducted comprehensive investigation of *Shigella* serotype distribution and antimicrobial resistance pattern in Xinjiang, identifying 458 *Shigella* isolates between 2008 to 2014. *Shigella flexneri* was identified as predominant species, and several *S*. *flexneri* serotypes were isolated, including atypical serotypes 1c, 2c, and 4s. Dominant *S*. *flexneri* serotypes were 2a, 1b, 2b, and Xv, different from those generally dominant in China. A hybrid serotype pattern was observed, which included the major Chinese serotypes (2a, Xv) and those predominant in Pakistan (1b, 2b). *Shigella sonnei* was shown to have a lower frequency compared with that generally observed in China, but an increasing trend of infections associated with this pathogen was observed. Furthermore, a high frequency of drug resistance and different *Shigella* antimicrobial resistance patterns were demonstrated as well, including very severe resistance phenotypes, such as multidrug resistance and resistance to frontline antibiotics. Seventy-five cephalosporin-resistant *Shigella* isolates were frequently identified with the resistance determinants that can undergo horizontal transfer, such as *bla*_OXA_, *bla*_TEM_, *bla*_CTX-M_, and integrons, facilitating the development of cephalosporin resistance among *Shigella* subtypes. Additionally, genetic analyses demonstrated that all 86 quinolone-resistant *S*. *flexneri* isolates possess 3–4 mutation sites in quinolone resistance-determining regions, primarily contributing to their resistance to quinolone. However, *S*. *sonnei* isolates were not shown to be quinolone resistant. Co-resistance to cephalosporins and quinolones was detected in 17 *S*. *flexneri* isolates, and these isolates were additionally multidrug resistant and carried β-lactamase genes and quinolone-resistance determinants. As is demonstrated in this study, dominant serotypes of *Shigella* were distributed in unique trend with dangerous drug resistance patterns. Novel strategies are urgently required to prevent the development of drug resistance among diarrhea-inducing pathogens.

## Introduction

*Shigella* spp. are recognized as important causative agents of diarrheal diseases in humans [1–4]. *Shigella* infections are considered the major public health burden worldwide, especially in the undeveloped and developing countries, and regions with poor sanitary conditions, with an estimated 167 million cases and about 1 million deaths annually; [5], and children under five are the most affected group [6]. Despite the improvements in economic and health conditions, shigellosis remains one of the top four notifiable infectious diseases, with half a million cases in China [7–9].

*Shigella* genus comprises four species, including *Shigella flexneri*, *Shigella dysenteriae*, *Shigella boydii*, and *Shigella sonnei* [10], and the distribution of these species and their serotypes shows distinct regional variations. *S*. *flexneri* has primarily been the epidemic species, caused diarrhea in developing countries, while *S*. *sonnei* has been prevalent in the developed countries [11]. However, *S*. *sonnei* prevalence has demonstrated an increasing trend in some Asian countries recently [12–15], together with the economic development. Furthermore, several serologically atypical isolates of *S*. *flexneri* were identified in recent studies [16–18], and these *Shigella* subgroup and serotype variations may lead to difficulties in the prevention and treatment of *Shigella* infection. The emergence and dissemination of antimicrobial resistance (AMR) aggravate *Shigella* prevalence. A trimethoprim/sulfamethoxazole-resistant *Shigella* isolate was first reported in Japan [19,20], followed by the emergence of diverse resistant *Shigella* types [21,22]. Previous studies reported a frequent resistance to some of the commonly used antibiotics, such as ampicillin and tetracyclines, worldwide [23–25]. Recently, the resistance to quinolones and cephalosporins was reported as well [12,26]. China is currently facing an increased risk of AMR dissemination among different types of intestinal pathogens. The antibiotic-resistant *Shigella* isolates have been identified throughout China [1,7,16,26], aggravating the challenges associated with the treatment and prevention of shigellosis.

As a region located in northwestern China, Xinjiang is bordered by eight countries. Historically, an important trading route, the ancient Silk Road, passed through Xinjiang, leading to the trans-regional migration of different populations. Today, increased contacts between China and other Asian or European countries, due to the proposed development of the Belt and Road, will occur through Xinjiang once more (http://hkmb.hktdc.com/en/1X0A5D5S/hktdc-research/Xinjiang-A-Core-Component-of-Belt-and-Road). The increase in the human economic activity may allow faster dissemination of infectious diarrhea pathogens and AMR. Additionally, a comprehensive survey of locally-present infectious pathogens is important to maintain the biosecurity of fast-growing economies. However, the prevalence and characterization of *Shigella* in Xinjiang has not been thoroughly analyzed before [27,28]. To improve the prevention and treatment of potential future *Shigella* epidemics, we performed detailed analyses of the prevalence and AMR patterns of *Shigella* isolates in Xinjiang, China. We analyzed the variations in *Shigella* species and serotype trends, characterized the AMR profile of these strains, and identified dominant antibiotic-resistant determinants of these isolates.

## Material and methods

### Bacterial isolation and *Shigella* serotyping

During routine surveillance of bacillary dysentery, fecal samples were collected from patients with diarrhea between 2008 and 2014 in Xinjiang. To isolate *Shigella* strains, the samples were directly streaked on *Salmonella-Shigella* agar (*SS* agar) (Beijing Land Bridge Technology CO., LTD, China) and incubated at 37°C for 16–22 h. The resultant *Shigella*-like colonies were steaked on the *SS* agar again and continually incubated at 37°C for 16–22 h. Following the second incubation, *Shigella* colonies were picked and streaked on Luria-Bertani agar plates, followed by incubation at 37°C for 16–22 h, after which these isolates were identified with API 20E test strips (bioMérieux SA, Marcy l’Etoile, France), according to the manufacturer’s instructions. The serotyping of *Shigella* isolates was performed using Shigella Antisera (Denka Seiken, Tokyo, Japan) and monoclonal antibody reagents (MASF IV-1 and MASF IV-2, Reagensia AB, Stockholm, Sweden). Written informed consents were obtained from patients or their guardians. All experiments were approved and authorized by the Ethics Committees of the Institute of Disease Prevention and Control, People’s Liberation Army, China.

### Antimicrobial susceptibility testing

Antimicrobial susceptibility testing was performed using the automated broth microdilution (Sensititre; Thermo Fisher Scientific, USA). Minimum inhibitory concentrations (MICs) of 21 antimicrobial agents were determined, including those of piperacillin, ampicillin, ticarcillin, ticarcillin/clavulanic acid, ceftazidime, ceftriaxone, cefepime, cefoperazone, cefazolin, cefoxitin, imipenem, nitrofurantoin, levofloxacin, norfloxacin, tetracycline, tobramycin, gentamicin, amikacin, aztreonam, chloramphenicol, and trimethoprim/sulfamethoxazole. Each isolate was identified as resistant or susceptible to each antibiotic, according to the cut-offs defined by the Clinical and Laboratory Standards Institute (CLSI 2017) [29]. *Escherichia coli* ATCC 25922 strain was used as the susceptible control strain.

### Detection of AMR determinants and integrons

Total DNA was extracted from all *Shigella* isolate using the TIANamp Bacterial DNA kit (Tiangen Biotech, Beijing, China). β-lactamase genes (*bla*_CTX-M_, *bla*_TEM_, *bla*_OXA_, *bla*_VIM_ and *bla*_NDM_) [30–32], quinolone resistance-determining regions (QRDRs) (*gyrA*, *gyrB*, *parC*, and *parE*) [32], plasmid-mediated quinolone resistance (PMQR) genes (*qnrA*, *qnrB*, *qnrD*, *qnrS*, and *aac(6’)-Ib-cr*) [31–33], and the variable regions of class 1 and class 2 integrons [34,35] were screened using PCR and the primers listed in S1 Table. The resulting PCR products were sequenced by BGI, Beijing, China, and the obtained data were edited using DNAstar (DNAstar Inc., Madison, WI, USA) and analyzed using Basic Local Alignment Search Tool in NCBI.

### Statistical analysis

Differences in the AMR rates and the frequency of AMR determinants between *Shigella* species were analyzed using χ^2^ test. Variations in *Shigella* prevalence with time were analyzed using linear regression. All statistical analyses were performed using Graph Pad 7.0 software, and P < 0.05 was considered statistically significant.

## Results

### Identification of isolates and distribution of *Shigella* spp

**Fig 1:**
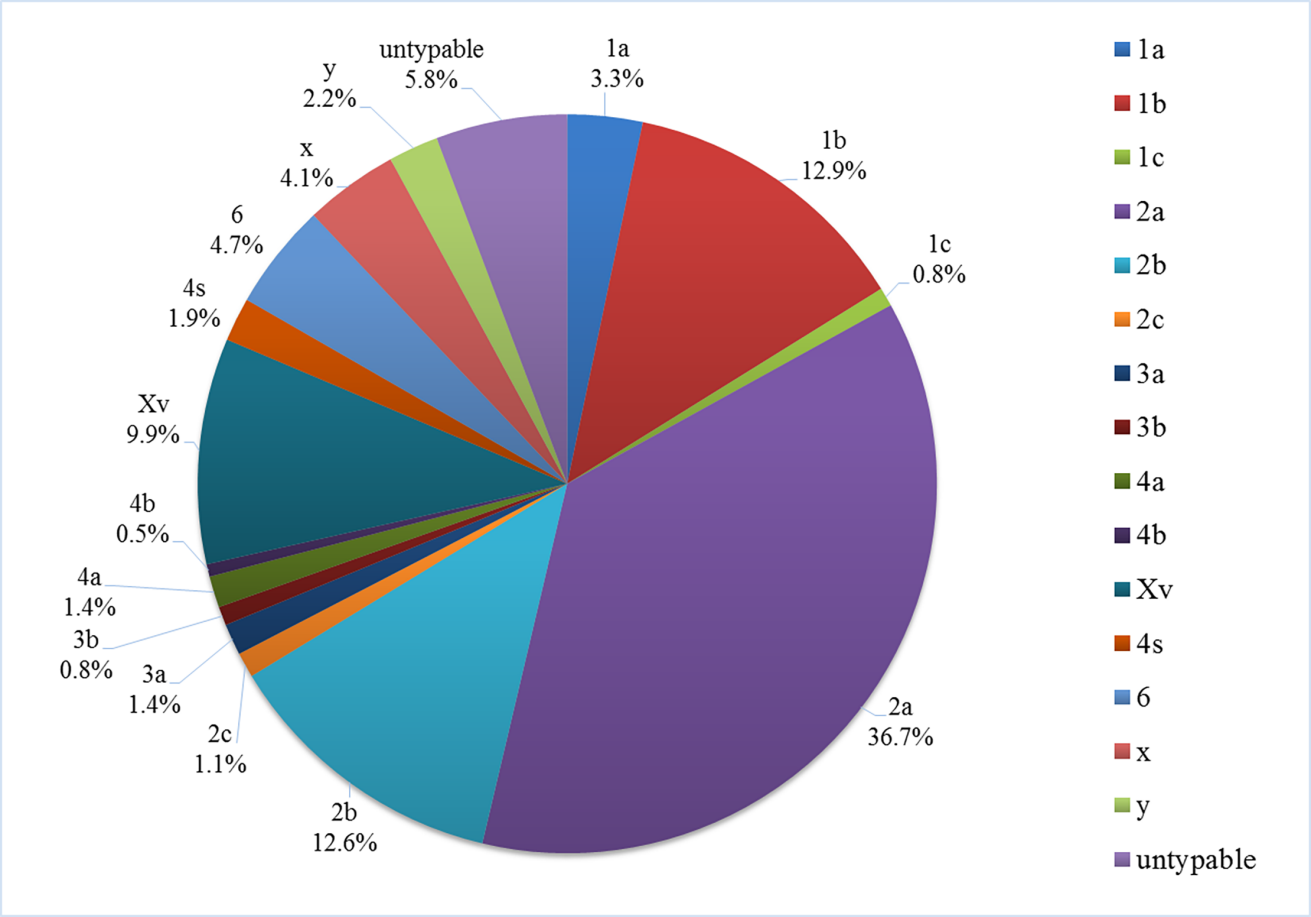
*S*. *flexneri* serotype distribution. Our analyses showed that 2a serotype was the most frequent among 19 identified serotypes. Serotype 2a, 2b, 1b, Xv represent the dominant serotypes, while others were rarely detected.

**Fig 2:**
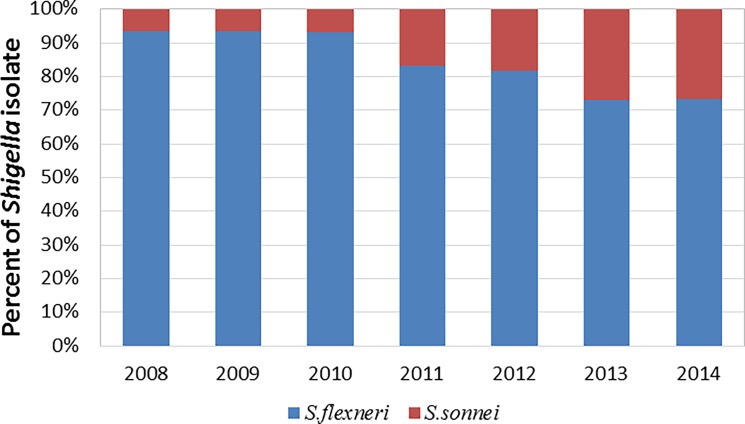
Trends in *Shigella* prevalence in isolates collected in Xinjiang between 2008 and 2014. Variations in the frequencies of *S*. *flexneri* and *S*. *sonnei* with time are presented. In each column, frequencies of *S*. *flexneri* and *S*. *sonnei* are shown.

All *Shigella* isolates in our study were collected from sentinel hospitals in 12 cities or prefectures (Urumqi, Karamay, Kashgar Prefecture, Aksu Prefecture, Hotan Prefecture, Turpan Prefecture, Hami Prefecture, Tacheng Prefecture, Altay Prefecture, Bortala Mongol Autonomous Prefecture, Changji hui autonomous prefecture, Bayingolin Mongol Autonomous Prefecture, Ili Kazakh Autonomous Prefecture). Among all cases, male patient accounted for 55.0%, female accounted for 45.0%; children or teenagers under 18 accounted for 32.9%, adult accounted for 67.1%. In total, we collected 458 *Shigella* isolates between 2008 and 2014 (Table 1). Of these, 365 (79.7%) were identified as *S*. *flexneri* isolates, and 93 (20.3%) were identified as *S*. *sonnei* isolates. *S*. *boydii* and *S*. *dysenteriae* presence was not detected. All collected *S*. *flexneri* isolates belonged to one of at least 16 serotypes (Fig 1), while serotype 2a (134, 36.7%) was identified as the major *S*. *flexneri* serotype detected during 7 years of surveillance, followed by 1b (47, 12.9%), 2b (46, 12.6%), and Xv (36, 9.9%) (Fig 1). Some atypical serotypes were identified, including three 1c isolates, four 2c isolates, and seven 4s isolates. Consistently, *S*. *flexneri* was shown to be the dominant *Shigella* species every year, while *S*. *sonnei* isolates accounted for less than 30% of samples (Fig 2). However, we observed a change in this trend, with the percentage of *S*. *sonnei* among the isolates showing the tendency to increase with time (linear regression analysis, P < 0.05) (S1 Fig). *S*. *sonnei* species, together with the dominant *S*. *flexneri* serotypes (2a, 1b, 2b, and Xv), were shown to constitute the majority of *Shigella* isolates in Xinjiang, with the frequency of above 70% each year (Table 1).

**Table 1:** The prevalence of *Shigella* in Xinjiang between 2008 and 2014.

Species/ serotype	Number (%) of isolates							
	2008 (n = 15)	2009 (n = 46)	2010 (n = 30)	2011 (n = 41)	2012 (n = 91)	2013 (n = 150)	2014 (n = 85)	Total (n = 458)
***S*. *flexneri***	14 (93.3%)	43 (93.5%)	28 (93.3%)	34 (82.9%)	75 (82.4%)	109 (72.7%)	62 (72.9%)	365 (79.7%)
1a	0	0	2 (6.7%)	1 (2.4%)	3 (3.3%)	5 (3.3%)	1 (1.2%)	12 (2.6%)
1b	3 (2)	3 (6.5%)	7 (23.3%)	7 (17.1%)	6 (6.6%)	13 (8.7%)	8 (9.4%)	47 (10.3%)
1c	0	1 (2.2%)	2 (6.7%)	0	0	0	0	3 (0.7%)
2a	5 (33.3%)	26 (56.5%)	6 (20%)	15 (36.6%)	22 (24.2%)	37 (24.7%)	23 (27.1%)	134 (29.3%)
2b	2 (13.3%)	4 (8.7%)	5 (16.7%)	7 (17.1%)	8 (8.8%)	15 (10%)	5 (5.9%)	46 (10.0%)
2c	0	0	0	1 (2.4%)	1 (1.1%)	2 (1.3%)	0	4 (0.9%)
3a	1 (6.7%)	1 (2.2%)	0	2 (4.9%)	0	0	1 (1.2%)	5 (1.1%)
3b	0	0	0	0	1 (1.1%)	1 (0.7%)	1 (1.2%)	3 (0.7%)
4a	0	0	0	0	1 (1.1%)	2 (1.3%)	2 (2.4%)	5 (1.1%)
4b	0	0	0	0	0	2 (1.3%)	0	2 (0.4%)
Xv	2 (13.3%)	0	2 (6.7%)	1 (2.4%)	13 (14.3%)	9 (6.0%)	9 (10.6%)	36 (7.9%)
4s	0	0	0	0	0	7 (4.7%)	0	7 (1.5%)
6	0	1 (2.2%)	1 (3.3%)	0	3 (3.3%)	7 (4.7%)	5 (5.9%)	17 (3.7%)
x	1 (6.7%)	1 (2.2%)	1 (3.3%)	0	4 (4.4%)	6 (4.0%)	2 (2.4%)	15 (3.3%)
y	0	1 (2.2%)	0	0	5 (5.5%)	1 (0.7%)	1 (1.2%)	8 (1.7%)
Untypable	0	5 (10.9%)	2 (6.7%)	0	8 (8.8%)	2 (1.3%)	4 (4.7%)	21 (4.6%)
*S*. *flexneri* 2a, 1b, 2b and Xv	12 (80.0%)	33 (71.7%)	20 (66.7%)	30 (73.2%)	49 (53.8%)	74 (49.3%)	45 (52.9%)	263 (57.4%)
***S*. *sonnei***	1 (6.7%)	3 (6.5%)	2 (6.7%)	7 (17.1%)	16 (17.6%)	41 (27.3%)	23 (27.1%)	93 (20.3%)
*S*. *sonnei*+ *S*. *flexneri* 2a, 1b, 2b and Xv	13 (86.7%)	36 (78.3%)	22 (73.3%)	37 (90.2%)	65 (71.4%)	115 (76.7%)	68 (80.0%)	356 (77.7%)

### *Shigella* AMR in Xinjiang

Antimicrobial susceptibility testing demonstrated that *S*. *flexneri* isolates are frequently resistant to ampicillin (94.0%), followed by the resistance to ticarcillin (92.9%), tetracycline (88.8%), chloramphenicol (87.7%), and trimethoprim/sulfamethoxazole (44.4%) (Table 2). Additionally, *S*. *flexneri* isolates showed a significantly higher resistance to several antimicrobials, including ceftazidime, levofloxacin, norfloxacin, ampicillin, ticarcillin, chloramphenicol, and ticarcillin/clavulanic acid, compared with that observed for the *S*. *sonnei* strains (χ^2^ analysis, P < 0.05). Furthermore, 57 (15.6%) *S*. *flexneri* isolates were shown to be resistant to cephalosporins, including cefazolin (15.3%), ceftriaxone (14.5%), cefoperazone (12.3%), ceftazidime (2.2%), and cefoxitin (0.5%). Moreover, 86 (23.6%) quinolone-resistant *S*. *flexneri* isolates (norfloxacin, 23.6%; levofloxacin, 8.5%) were detected in Xinjiang.

**Table 2:** Antimicrobial resistance of *Shigella* isolates recovered from patients with diarrhea in Xinjiang, China between 2008 and 2014.

Antimicrobial agents	Total (n = 458)		*S*. *flexneri* (n = 365)		*S*. *sonnei* (n = 93)		χ2	P
	No	%	No	%	No	%		
**Cephems**								
FEP	0	0	0	0	0	0	/	/
FOX	2	0.4	2	0.5	0	0	0.5118	P = 0.4744
CAZ	8	1.7	8	2.2	0	0	8.601,	P = 0.0034
CFP	63	13.8	45	12.3	18	19.4	3.084	P = 0.0791
CRO	53	11.6	53	14.5	18	19.4	1.322	P = 0.2502
CFZ	74	16.2	56	15.3	18	19.4	0.8808	P = 0.3480
**Fluoroquinolones**								
LEV	31	6.8	31	8.5	0	0	8.472	P = 0.0036
NOR	86	18.8	86	23.6	0	0	26.98	P < 0.0001
**Carbapenems**								
IMP	0	0.0	0	0	0	0	/	/
**Penicillins**								
PIP	66	14.4	45	12.3	21	22.6	6.316	P = 0.0120
TIC	399	87.1	339	92.9	60	64.5	53.12	P < 0.0001
AMP	403	88.0	343	94	60	64.5	60.86	P < 0.0001
**Aminoglycosides**								
AK	1	0.2	1	0.3	0	0	0.2554	P = 0.6133
TO	11	2.4	9	2.5	2	2.1	0.03142	P = 0.8593
GN	57	12.4	16	4.4	41	44.1	107.2	P < 0.0001
**Monobactams**								
ATM	33	7.2	18	4.9	15	16.1	13.9	P = 0.0002
**Nitrofurans**								
NIT	0	0.0	0	0	0	0	/	/
**Tetracyclines**								
TE	407	88.9	324	88.8	83	89.2	0.01727	P = 0.8954
**Phenicols**								
C	322	70.3	320	87.7	2	2.1	259.7	P < 0.0001
**β-Lactam/β-lactamase inhibitor combinations**								
TIM	35	7.6	34	9.3	1	1.1	7.13	P = 0.0076
**Folate pathway inhibitors**								
SXT	249	54.4	162	44.4	87	93.5	72.21	P < 0.0001

AK, amikacin; AMP, ampicillin; ATM, aztreonam; CFZ, cefazolin; FEP, cefepime; CFP, cefoperazone; FOX, cefoxitin; CAZ, ceftazidime; CRO, ceftriaxone; C, chloramphenicol; GEN, gentamicin; IPM, imipenem; LEV, levofloxacin; NIT, nitrofurantoin; NOR, norfloxacin; PIP, piperacillin; SXT, trimethoprim/sulfamethoxazole; TE, tetracycline; TIC, ticarcillin; TIM, ticarcillin/clavulanic acid; TO, tobramycin.

In contrast, *S*. *sonnei* species was shown to be the most resistant to trimethoprim/sulfamethoxazole (93.5%), followed by the resistance to tetracycline (89.2%), ticarcillin (64.5%), ampicillin (64.5%), gentamicin (44.1%) (Table 2). Furthermore, *S*. *sonnei* showed a significantly higher resistance rates to some antibiotics, including piperacillin, gentamicin, aztreonam, and trimethoprim/sulfamethoxazole, compared with those obtained for *S*. *flexneri* isolates (χ^2^, P < 0.05). Additionally, *S*. *sonnei* isolates showed resistance to cephalosporins, including cefazolin (19.4%), ceftriaxone (19.4%), and cefoperazone (19.4%). None of these isolates was resistant to other cephalosporins, such as cefepime, cefoxitin, and ceftazidime, and to quinolones (levofloxacin and norfloxacin; Table 2).

Furthermore, 330 (90.4%) of 365 *S*. *flexneri* isolates and 59 (63.4%) of 93 *S*. *sonnei* isolates were shown to be multidrug resistant (MDR, resistant to three or more CLSI classes of antimicrobials) (Table 3 and S2 Table). *S*. *flexneri* species showed a significantly higher frequency of MDR than *S*. *sonnei* species (χ^2^, P < 0.05). *S*. *flexneri* exhibited MDR to at most 7 classes of antimicrobial and *S*. *sonnei* showed MDR to at most 5 classes of antimicrobials. Besides, there were some *S*. *flexneri* isolates with important MDR phenotypes, including 136 (37.3%) *S*. *flexneri* isolates resistant to at least ampicillin, chloramphenicol, tetracycline and trimethoprim/sulfamethoxazole; 38 (10.4%) *S*. *flexneri* isolates resistant to at least ampicillin, chloramphenicol, tetracycline, trimethoprim/sulfamethoxazole and norfloxacin; 22 (6%) *S*. *flexneri* isolates resistant to at least ampicillin, chloramphenicol, tetracycline, trimethoprim/sulfamethoxazole and ceftriaxone; 9 (2.5%) *S*. *flexneri* isolates resistant to at least ampicillin, chloramphenicol, tetracycline, trimethoprim/sulfamethoxazole, norfloxacin and ceftriaxone. Notably, these important MDR phenotypes have not been observed in *S*. *sonnei* isolates.

**Table 3:** Antimicrobial resistance profiles of *Shigella* isolates in Xinjiang between 2008 and 2014.

Antibiotic*	Number of isolates (%)	
	*S*. *flexneri* (n = 365)	*S*. *sonnei* (n = 93)
No resistance detected	12 (3.3)	3 (3.2)
Resistant ≥ 1 CLSI class	353 (96.7)	90 (96.8)
Resistant ≥ 2 CLSI classes	349 (95.6)	90 (96.8)
Resistant ≥ 3 CLSI classes	330 (90.4)	59 (63.4)
Resistant ≥ 4 CLSI classes	200 (54.8)	53 (57.0)
Resistant ≥ 5 CLSI classes	62 (17.0)	14 (15.1)
Resistant ≥ 6 CLSI classes	21 (5.8)	0
Resistant = 7 CLSI classes	8 (2.2)	0
Cephalosporin	57 (15.6)	18 (19.4)
Quinolones	86 (23.6)	0
Cephalosporin and quinolones	17 (4.7)	0
At least ACTT/S	136 (37.3)	0
At least ACTT/SNOR	38 (10.4)	0
At least ACTT/SCRO	22 (6.0)	0
At least ACTT/SCRONOR	9 (2.5)	0

*A, ampicillin; C, chloramphenicol; CRO, ceftriaxone; NOR, norfloxacin; T, tetracycline; T/S, trimethoprim/sulfamethoxazole.

### Detection of AMR determinants and integrons

A total of 57 (15.6%) *S*. *flexneri* and 18 (19.4%) *S*. *sonnei* isolates showing high-level resistance to cephalosporin were selected to detect the presence of antibiotic-resistance determinant genes and integrons (Table 4). Rates of *bla*_OXA-1_, *bla*_TEM-1,_ and *bla*_CTX-M_ expression among the selected *S*. *flexneri* isolates were 82.5%, 61.4%, and 89.5%, respectively. Moreover, 48 *S*. *flexneri* isolates contained the CTX-M gene, including 23 (40.4%) isolates containing CTX-M-1 group genes, 29 (50.9%) isolates with CTX-M-9 group genes, and 4 (7.0%) isolates containing both group genes. No expression of *bla*_VIM_ and *bla*_NDM_ was observed in the tested isolates. Furthermore, the majority of tested *S*. *flexneri* isolates was shown to harbor integrons, including 49 (86.0%) isolates that were shown to harbor class 1 integrons and 51 (89.4%) that harbored class 2 integrons (Table 4). In contrast to the *S*. *flexneri* isolates, all selected *S*. *sonnei* isolates were negative for *bla*_OXA-1_ expression, while only 38.9% expressed *bla*_TEM_ (Table 4). The expression rate of class 1 integrons detected in *S*. *sonnei* isolates (5.6%) was considerably lower than that in the *S*. *flexneri* isolates (86.0%), while the rate of isolates expressing class 2 integrons reached 83.3%. All 18 tested cephalosporin-resistant *S*. *sonnei* isolates were demonstrated to harbor *bla*_CTX-M_ genes, including 14 (77.8%) isolates with CTX-M-1 group genes, nine (50%) isolates containing CTX-M-9 group genes, and six (33.3%) isolates carrying genes from both of these groups (Table 4). Among cephalosporin-resistant isolates, a higher frequency of isolates carrying both CTX-M-1 and CTX-M-9 group genes was detected among *S*. *sonnei* isolates, compared with that observed among *S*. *flexneri* isolates (χ^2^ analysis, P < 0.05).

**Table 4:** Antimicrobial resistance determinants in 75 cephalosporin-resistant *Shigella* isolates and 86 quinolone-resistant *Shigella* isolates.

Resistant determinant	Number of isolates (%)		
**Determinants of cephalosporin resistance**	***S*. *flexneri* (n = 57)**	***S*. *sonnei* (n = 18)**	**Total (n = 75)**
*bla*_VIM_	0	0	0
*bla*_NDM_	0	0	0
*bla*_OXA_	47 (82.5)	0	47 (62.7)
*bla*_TEM_	35 (61.4)	7 (38.9)	42 (56.0)
*bla*_CTX-M_	51 (89.5)	18 (100%)	69 (92.0)
CTX-M-1 group	23 (40.4)	14 (77.8)	37 (49.3)
*bla*_CTX-M-3_	3 (5.3)	0 (0)	3 (4.0)
*bla*_CTX-M-15_	10 (17.5)	7 (38.9)	17 (22.7)
*bla*_CTX-M-28_	2 (3.5)	0 (0)	2 (2.7)
*bla*_CTX-M-55_	6 (10.5)	7 (38.9)	13 (17.3)
*bla*_CTX-M-64_	2 (3.5)	0 (0)	2 (2.7)
CTX-M-9 group	29 (50.9)	4 (22.2)	33 (44.0)
*bla*_CTX-M-14_	26 (45.6)	4 (22.2)	30 (40.0)
*bla*_CTX-M-24_	3 (5.3)	0 (0)	3 (4.0)
Both CTX-M-1 and CTX-M-9 group	4 (7.0)	6 (33.3)	10 (13.3)
*intl1*	49 (86.0)	1 (5.6)	50 (66.7)
*intl2*	51 (89.5)	15 (83.3)	66 (88.0)
*hep74-51*	49 (86.0)	17 (94.4)	66 (88.0)
**Determinants of resistance to quinolones**	***S*. *flexneri* (n = 86)**	***S*. *sonnei* (n = 0)**	**Total (n = 86)**
QRDRs mutations	86 (100)	0	86 (100)
*gyrA*	86 (100)	0	86 (100)
*Ser83Leu*	86 (100)	0	86 (100)
*Asp87Asn*	62 (72.1)	0	62 (72.1)
*Asp87Gly*	16 (18.6)	0	16 (18.6)
*His211Tyr*	82 (95.3)	0	82 (95.3)
*gyrB*	0	0	0
*parE*	0	0	0
*parC*	86 (100)	0	86 (100)
*Ser80Ile*	86 (100)	0	86 (100)
Triple (or more) mutations	86 (100)	0	86 (100)
Four mutations	74 (86.0)	0	74 (86.0)
PMQR genes	3 (3.5)	0	3 (3.5)
*qnrB*	1 (1.2)	0	1 (1.2)
*qnrS*	2 (2.3)	0	2 (2.3)
*acc (6’)-Ib-cr*	0	0	0

Moreover, in this study, we identified 86 *S*. *flexneri* isolates resistant to quinolones (levofloxacin or norfloxacin) (Table 4). QRDR mutations were detected in three amino acids of the *gyrA* gene (Ser83Leu, Asp87Asn/Gly, His211Tyr) and one amino acid of the *parC* gene (Ser80Ile). All 86 isolates were shown to harbor Ser83Leu mutations in the *gyrA* and Ser80Ile in the *parC* genes. Additionally, 62 isolates were shown to carry Asp87Asn mutation in the *gyrA* gene, 16 isolates had Asp87Gly mutation in the *gyrA* and 82 isolates had His211Tyr mutation in the *gyrA* gene. No point mutations in the *gyrB* and *parE* genes were observed. All 86 quinolone-resistant isolates were identified to carry at least three types of QRDR mutations, and 74 (86.0%) isolates were shown to harbor four types of QRDR mutations. In contrast, only three isolates were shown to harbor PMQR gene mutations (one in *qnrB*, two in *qnrS*), and no isolates were positive for *acc(6′)-Ib-cr*.

Notably, 17 *Shigella* isolates were resistant to cephalosporins and quinolones simultaneously (Table 5), and these were the MDR *S*. *flexneri* strains. Other than the resistance to cephalosporins and quinolones, the resistance to penicillins (17, 100%) was one of the most frequent types of resistance, followed by the resistance to tetracyclines (16, 94.1%), amphenicols (15, 88.2%), folate pathway inhibitors (13, 76.5%), and monobactams (6, 35.3%). Molecular analysis of the resistance determinants in 17 isolates, demonstrated the expression of *bla*_OXA-1_ in 16 isolates, *bla*_TEM-1_ in 14 isolates, and *bla*_CTX-M_ in 15 isolates. Quinolone resistance of all 17 isolates was shown to be mediated by the mutations in QRDRs of *gyrA* and *parC*, since no isolate was shown to have PMQR gene mutations. Integrons and gene cassette arrays were identified in a number of isolates, including 17 isolates shown to carry integrons, 16 isolates with class 1 integrons, and 16 with class 2 integrons.

**Table 5:** Antimicrobial resistance profiles and resistance determinants of 17 *Shigella* isolates co-resistant to cephalosporin and quinolone.

Isolate	Antimicrobial resistance profile	β-Lactamases gene			QRDRs mutation		PMQR gene	Integron		
		*bla*_OXA-1_	*bla*_TEM-1_	*bla*_CTX-M_	*gyrA*	*parC*	*qnrB/qnrS*	*IntI1*	*IntI2*	hep74-51
XJSF20	CRO/CFZ/TIC/AMP/NOR/TE/C/STX	+	+	*bla*_CTX-M-15_	S83L, D87N, H211Y	S80I	-	+	+	+
XJSF22	CRO/CFP/CFZ/TIC/AMP/PIP/NOR/TE/ATM/C/STX	+	+	*bla*_CTX-M-55_	S83L, D87G, H211Y	S80I	-	+	+	+
2010048	CRO/CFP/CFZ/TIC/AMP/PIP/NOR/TE/ATM/C/STX	+	+	*bla*_CTX-M-55_	S83L, D87N, H211Y	S80I	-	+	+	+
2011109	CRO/CFP/CFZ/TIC/TIM/AMP/PIP/NOR/GN/TE/ATM/C/STX	+	+	*bla*_CTX-M-55_	S83L, D87G, H211Y	S80I	-	+	+	+
2012064	CAZ/CRO/CFP/CFZ/TIC/AMP/NOR/TE/C	+	+	*bla*_CTX-M-15_	S83L, D87G, H211Y	S80I	-	+	+	+
2012076	CRO/CFP/CFZ/TIC/TIM/AMP/PIP/LEV/NOR/TE/ATM/C/STX	+	+	*bla*_CTX-M-15_	S83L, H211Y	S80I	-	+	+	+
2012085	CFZ/TIC/TIM/AMP/NOR/TE/C/STX	+	+	-	S83L, D87N, H211Y	S80I	-	+	+	+
2012131	CRO/CFP/CFZ/TIC/AMP/PIP/NOR/TE/ATM/C	+	+	*bla*_CTX-M-55_	S83L, D87G, H211Y	S80I	-	+	+	+
2012136	CRO/CFP/CFZ/TIC/TIM/AMP/PIP/LEV/NOR/C	+	+	*bla*_CTX-M-14_	S83L, D87N, H211Y	S80I	-	+	+	+
2012262	CRO/CFP/CFZ/TIC/TIM/AMP/PIP/NOR/TE/C	+	+	-	S83L, D87G, H211Y	S80I	-	+	+	+
2013269	CAZ/CRO/CFP/CFZ/TIC/TIM/AMP/PIP/LEV/NOR/TE/ATM/C/STX	+	+	*bla*_CTX-M-15_	S83L, D87N, H211Y	S80I	-	+	+	+
2013398	CRO/CFP/CFZ/TIC/TIM/AMP/PIP/LEV/NOR/GN/TE/C/STX	+	+	*bla*_CTX-M-14_	S83L, H211Y	S80I	-	+	+	+
2013416	CRO/CFP/CFZ/TIC/AMP/PIP/NOR/TE/ATM/C/STX	+	+	*bla*_CTX-M-14, 64_	S83L, D87N, H211Y	S80I	-	+	+	+
2014104	CRO/CFP/CFZ/TIC/AMP/PIP/LEV/NOR/GN/TE/ATM/STX	-	-	*bla*_CTX-M-15_	S83L, D87N, H211Y	S80I	-	+	-	-
2014331	CRO/CFP/CFZ/TIC/AMP/PIP/NOR/TE/ATM/C/STX	+	+	*bla*_CTX-M-15_	S83L, D87N, H211Y	S80I	-	+	+	+
2014351	CRO/CFZ/TIC/AMP/LEV/NOR/TE/C/STX	+	-	*bla*_CTX-M-14_	S83L, H211Y	S80I	-	-	+	+
2014366	CRO/CFZ/TIC/AMP/LEV/NOR/TE/C/STX	+	-	*bla*_CTX-M-15_	S83L, D87N, H211Y	S80I	-	+	+	+

AK, amikacin; AMP, ampicillin; ATM, aztreonam; CFZ, cefazolin; FEP, cefepime; CFP, cefoperazone; FOX, cefoxitin; CAZ, ceftazidime; CRO, ceftriaxone; C, chloramphenicol; GEN, gentamicin; IPM, imipenem; LEV, levofloxacin; NIT, nitrofurantoin; NOR, norfloxacin; PIP, piperacillin; SXT, trimethoprim/sulfamethoxazole; TE, tetracycline; TIC, ticarcillin; TIM, ticarcillin/clavulanic acid; TO, tobramycin.

## Discussion

Here, we demonstrated that the most frequent species of *Shigella* genus in Xinjiang in all years of our study was *S*. *flexneri*. Recently, the *S*. *sonnei* has shown increasing prevalence in China and even become the dominant species in southeast and central parts of China [36]. Furthermore, together with the economic growth in Xinjiang, an obvious rising trend in *S*. *sonnei* infections can be observed. The frequency of identified *S*. *sonnei* isolates altered from 6.7% in 2008 to 27.1% in 2014, which was, even considering the increase, shown to be below the average frequency of *S*. *sonnei* infections registered in China, where it was the dominant *Shigella* species (58.2% in 2011–2013) [36]. Various studies have concluded that *S*. *sonnei* prevalence increase with economic development [37]. As Xinjiang will continue to enhance its level of economy and sanitation, it is likely that *S*. *sonnei* species would become even more of a local public health concern. The design of vaccine candidate and shigellosis prevention strategy should consider this change of *Shigella* epidemics.

Considering serotypes, 2a and Xv were reported to be dominant *S*. *flexneri* serotypes in China [36], and 2a was reported to be dominant *S*. *flexneri* serotype in Xinjiang [27]. However, the results of our study further demonstrated that 2a serotype was still the most prevalent in Xinjiang but Xv serotype accounted for a smaller proportion of *S*. *flexneri* isolates, while 2a, 1b, 2b, and Xv represent the dominant serotypes detected between 2008 and 2014. Serotypes 1b and 2b are less frequent in China [36], but common in Pakistan [38], a country bordering Xinjiang. Therefore, the dominant *S*. *flexneri* serotypes distributed in Xinjiang likely represent the mixture of the major serotypes in China and adjacent countries.

Furthermore, we detected atypical serotypes 1c, 2c, and 4s in Xinjiang. Serotype-based vaccines are currently under development, as a promising strategy against *Shigella* epidemics [39]. A future *Shigella* vaccine potentially applied in Xinjiang should be developed in accordance considering the characteristic *Shigella* serotypes in this region, and future surveillance studies in Xinjiang should pay close attention not only to the newly emerged serotypes but the predominant subgroups in surrounding regions as well, in order to prevent potential *Shigella* epidemics caused by bacteria with novel or imported O-antigen types.

*Shigella* isolates demonstrated high levels of resistance to antibiotics. Both *S*. *flexneri* and *S*. *sonnei* revealed high AMR rate to some common used antibiotics like penicillins and tetracyclines. Thus, these older-generation drugs should not be contained in empirical therapy of shigellosis. Notably, 330 of 365 *S*. *flexneri* isolates and 59 of 93 *S*. *sonnei* isolates collected from Xinjiang showed MDR profiles. The high-level MDR frequency further restrict the choice of antibiotics in the clinical treatment of bacterial infections. Since the efficacy of older-generation antibiotics decreased due to the development of resistant strains, in a previous study, quinolones and third-generation cephalosporins were recommended as frontline antimicrobials for the empiric treatment of diarrhea-inducing pathogens [40]. However, in our study, 75 (16.38%) isolates were resistant to cephalosporin and 86 (18.77%) were resistant to norfloxacin. Additionally, 17 (3.71%) isolates were resistant to both cephalosporins and quinolones. Considering the currently used frontline antimicrobials, these resistance phenotypes threaten the effectiveness of therapy [41,42]. The mobility and dissemination of the resistant strains may increase in Xinjiang as it becomes an increasingly crucial region connecting China and other countries. Surveillance to *Shigella* in China revealed its high MDR frequency [7,16,17,26,43,44]. The high-level resistant pathogen could migrate along with increasing trans-regional human activities. Therefore, novel preventive strategies are urgently required to prevent the spreading of AMR among *Shigella* strains. Clinicians prescribing anti-infective therapies should be more cautious, since the unreasonable use of antibiotics may further accelerate the accumulation and spread of AMR [45–47].

The AMR profile differed between *S*. *flexneri* and *S*. *sonnei* strains. Specifically, *S*. *flexneri* revealed higher MDR levels, demonstrating also some specific and important MDR phenotypes. A proportion of *S*. *flexneri* strains (37.3%) was shown to be resistant to the combination of commonly used antibiotics, including ampicillin, chloramphenicol, and trimethoprim/sulfamethoxazole, which was not observed among *S*. *sonnei* isolates. Quinolone resistance and even co-resistance to cephalosporin and quinolone emerged in *S*. *flexneri* strains, while *S*. *sonnei* demonstrated the sensitivity to quinolone. As a long-term predominant species in China [48], the traditional antibiotic-associated selection of *S*. *flexneri* has been underway for decades, and by frontline antibiotics in recent years, which led to the development of the MDR strains and the resistance to cephalosporin and quinolone. Therefore, our results imply that the antibiotic therapy of choice may differ between two *Shigella* species. Treatment of *S*. *flexneri* infections may be more complicated than that of *S*. *sonnei*, and a drug susceptibility test should be performed immediately after diagnosing a patient with *S*. *flexneri* infection. Antibiotic abuse should be more controlled, in order to reduce the selection pressure on *S*. *flexneri* strains, but the quinolone treatment may represent a safer antibiotic choice if the infective pathogens are identified as *S*. *sonnei*.

In this study, we further elucidated the genetic background and mechanisms underlying *Shigella* resistance to cephalosporins and quinolones. Cephalosporin-resistant *S*. *flexneri* were shown to frequently express *bla*_CTX-M_, *bla*_OXA_, and *bla*_TEM_, three main genes conferring the resistance to cephalosporin [49,50]. All cephalosporin-resistant *S*. *sonnei* expressed *bla*_CTX-M_ as well. These genes were reported to be frequently encoded by plasmids [51–55], which facilitates the horizontal transfer of resistance to β-lactamase antibiotics [49,56]. Previous studies reported that class 1 and class 2 integrons may provide the resistance to other types of drugs and be responsible for the dissemination of AMR [35,57,58]. Almost 90% of the isolated *S*. *flexneri* strains were shown to harbor two integrons. The *intl2* was also frequently identified in the *S*. *sonnei* isolates (83.3%). These results indicate that the cephalosporin resistance determinants can actively disseminate among *Shigella* cells or transferr within microflora. The PMQR genes were reported to be always located in mobile genetic elements such as plasmids [59]. Here, the presence of *qnrS* and *qnrB* genes was detected at a very low level in quinolone-resistant *S*. *flexneri* in Xinjiang, while a number of QRDR mutations were identified, indicating that the mutations in QRDRs primarily underlie the resistance to quinolone in *Shigella* isolates investigated here. Different QRDR mutations confer various levels of resistance [60–62]. Notably, all quinolone-resistant isolates were shown to harbor at least three QRDR mutations, showing that their simultaneous presence may underlie the observed increase in the resistance to quinolones.

We analyzed and presented here the prevalence of *Shigella* species and serotypes in Xinjiang, China. *S*. *flexneri* was shown to be the dominant *Shigella* species, with a unique dominant serotype pattern (2a, 1b, 2b, Xv), which represents a hybrid pattern comprising serotypes prevalent in adjacent regions. High levels of AMR were observed, especially by *S*. *flexneri* isolates. Emergence of frequently observed MDR and resistance to frontline antibiotics can severely restrict the choice of antibiotic therapy used for the treatment of *Shigella* infections. Since unsafe sanitation conditions remain present in this region, food-borne or water-borne shigellosis epidemic will remain a significant public health concern in future [63,64]. Therefore, the prevalence, trends, and AMR patterns of *Shigella* species and serotypes in Xinjiang should be closely monitored, and novel strategies are urgently required to prevent the spreading of the AMR among *Shigella* strains.

## Supporting information

S1 Fig Variations in *S*. *sonnei* prevalence with time. An increasing trend in *S*. *sonnei* frequency among the *Shigella* isolates was observed between 2008 and 2014.
(TIF)

S1 Table Primers used for the PCR amplification of antibiotic resistance genes. (DOCX)

S2 Table MDR classes of *Shigella* strains isolated in Xinjiang, China. (DOCX)
