# Supplementary figures and images for: Dominant serotype distribution and antimicrobial resistance profile of Shigella spp. in Xinjiang, China
Source: PLoS One. 2018 Apr 3;13(4):e0195259. doi: 10.1371/journal.pone.0195259 (PMC5882154; doi:10.1371/journal.pone.0195259)

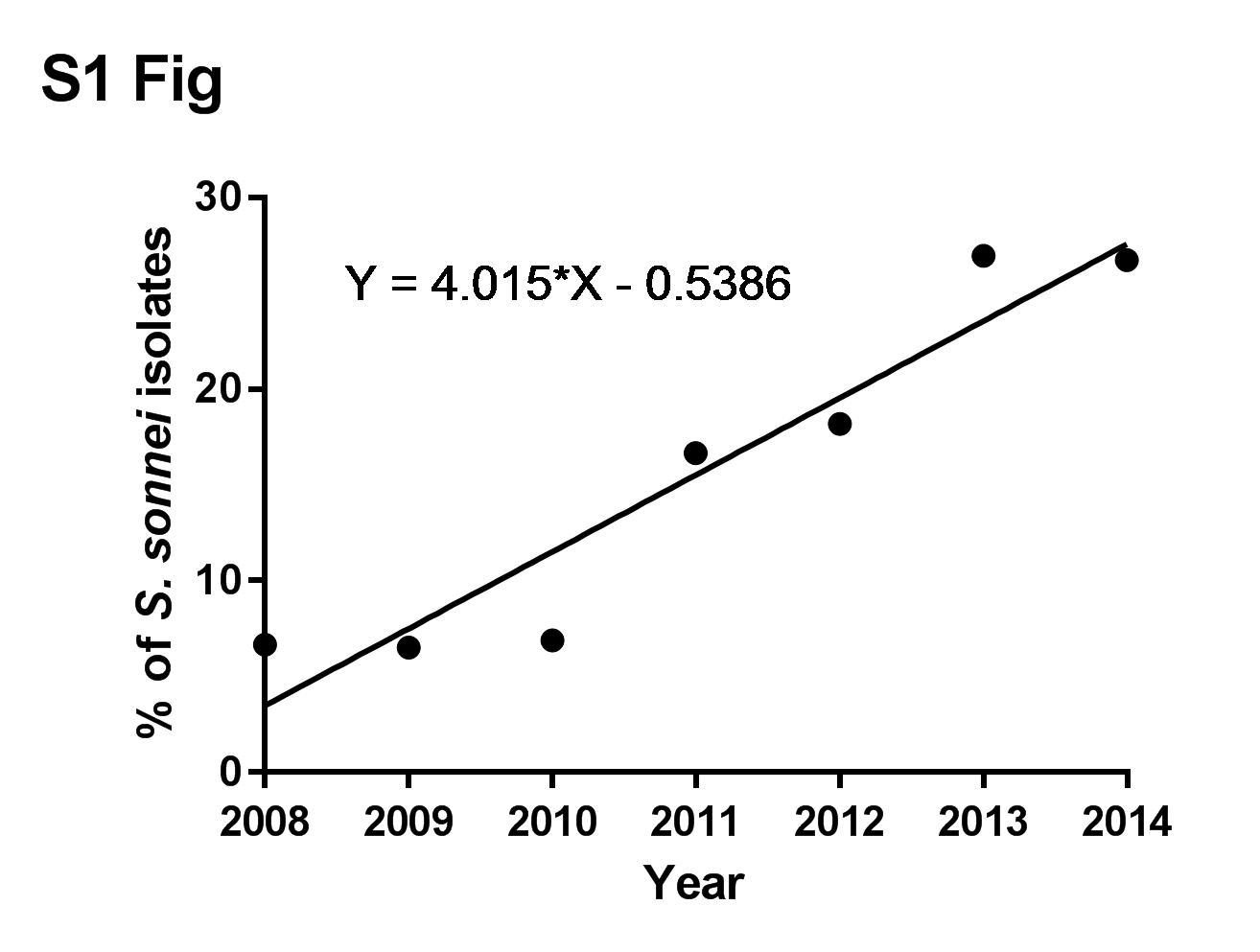

Supplement: S1 Fig — An increasing trend in S. sonnei frequency among the Shigella isolates was observed between 2008 and 2014. (TIF) [file pone.0195259.s001.tif]
