# Supplementary material for: Dominant serotype distribution and antimicrobial resistance profile of Shigella spp. in Xinjiang, China
Source: PLoS One. 2018 Apr 3;13(4):e0195259. doi: 10.1371/journal.pone.0195259 (PMC5882154; doi:10.1371/journal.pone.0195259)
Supplement: S1 Table — (DOCX) [file pone.0195259.s002.docx]

**S1 Table. Primers used for the PCR amplification of antibiotic resistance genes.**

| Primers | Nucleotide sequence (5′ to 3′) | Target | Length (bp) | Reference |
| --- | --- | --- | --- | --- |
| β-Lactamases | | | |  |
| *bla*_CTX-M-1_ group-F  *bla*_CTX-M-1_ group-R | GGTTAAAAAATCACTGCGTC  TTACAAACCGTCGGTGACGA | *bla*_CTX-M 1_ group | 873 | This study |
| *bla*_CTX-M-9_ group-F  *bla*_CTX-M-9_ group-R | AGAGTGCAACGGATGATG  CCAGTTACAGCCCTTCGG | *bla*_CTX-M 9_ group | 868 | This study |
| *bla*_CTX-M-2/8/25_ group-F  *bla*_CTX-M-2/8/25_ group-R | ACCGAGCCSACGCTCAA  CCGCTGCCGGTTTTATC | *bla*_CTX-M-2/8/25_ group | 221 | This study |
| *bla*_TEM_-F  *bla*_TEM_-R | ATGAGTATTCAACATTTCCG  CCAATGCTTAATCAGTGAGG | *bla*_TEM_ | 1080 | [[1](#_ENREF_1)] |
| *bla*_OXA_-F  *bla*_OXA_-R | ATTAAGCCCTTTACCAAACCA  AAGGGTTGGGCGATTTTGCCA | *bla*_OXA_ | 890 | [[2](#_ENREF_2)] |
| *bla*_VIM_-F3  *bla*_VIM_-R3 | AGTGGTGAGTATCCGACAG  ATGAAAGTGCGTGGAGAC | *bla*_VIM_ | 509 | [[3](#_ENREF_3)] |
| *bla*_NDM-1_-F  *bla*_NDM-1_-R | GTCTGGCAGCACACTTCCTA  TAGTGCTCAGTGTCGGCATC | *bla*_NDM-1_ | 515 | This study |
| Integrons | | | |  |
| *IntI1*-F2  *IntI1*-R2 | ACATGTGATGGCGACGCACGA  ATTTCTGTCCTGGCTGGCGA | *IntI1* | 569 | [[4](#_ENREF_4)] |
| *IntI2*-F3  *IntI2*-R3 | CACGGATATGCGACAAAAAGGT  GTAGCAAACGAGTGACGAAATG | *IntI2* | 789 | [[4](#_ENREF_4)] |
| *hep*58  *hep*59 | TCATGGCTTGTTATGACTGT  GTAGGGCTTATTATGCACGC | Class 1 integron variable region | variable | This study |
| *hep74*  *hep51* | CGGGATCCCGGACGGCATGCACGATTTGTA  GATGCCATCGCAAGTACGAG | Class 2 integron variable region | variable | [[2](#_ENREF_2)] |
| Chromosomal mutations-mediated quinolone resistance | | | |  |
| *gyrA*-F  *gyrA*-R | TACACCGGTCAACATTGAGG  TTAATGATTGCCGCCGTCGG | *gyrA* | 648 | [[5](#_ENREF_5)] |
| *gyrB*-F  *gyrB*-R | TGAAATGACCCGCCGTAAAGG  GCTGTGATAACGCAGTTTGTCCGGG | *gyrB* | 309 | [[5](#_ENREF_5)] |
| *parC*-F  *parC*-R | GTACGTGATCATGGACCGTG  TTCGGCTGGTCGATTAATGC | *parC* | 531 | [[5](#_ENREF_5)] |
| *parE*-F  *parE*-R | ATGCGTGCGGCTAAAAAAGTG  TCGTCGCTGTCAGGATCGATAC | *parE* | 290 | [[5](#_ENREF_5)] |
| Plasmid-mediated quinolone resistance | | | |  |
| *qnrA*-F3  *qnrA*-R3 | ATTTCTCACGCCAGGATTTG  GATCGGCAAAGGTYAGGTCA | *qnrA* | 516 | [[6](#_ENREF_6)] |
| *qnrB*-F  *qnrB*-R | GATCGTGAAAGCCAGAAAGG  ACGAYGCCTGGTAGTTGTCC | *qnrB* | 469 | [[6](#_ENREF_6)] |
| *qnrD*-F  *qnrD*-R | CGAGATCAATTTACGGGGAATA  AACAAGCTGAAGCGCCTG | *qnrD* | 656 | [[1](#_ENREF_1)] |
| *qnrS*-F  *qnrS*-R | ACGACATTCGTCAACTGCAA  TAAATTGGCACCCTGTAGGC | *qnrS* | 417 | [[6](#_ENREF_6)] |
| *aac(6')-Ib-cr*-F  *aac(6')-Ib-cr*-R | GCAACGCAAAAACAAAGTTAGG  GTGTTTGAACCATGTACA | *aac(6')-Ib-cr* | 560 | [[7](#_ENREF_7)] |

1. Tariq A, Haque A, Ali A, Bashir S, Habeeb MA, et al. (2012) Molecular profiling of antimicrobial resistance and integron association of multidrug-resistant clinical isolates of Shigella species from Faisalabad, Pakistan. Can J Microbiol 58: 1047-1054.

2. Ahmed AM, Furuta K, Shimomura K, Kasama Y, Shimamoto T (2006) Genetic characterization of multidrug resistance in Shigella spp. from Japan. J Med Microbiol 55: 1685-1691.

3. Galani I, Souli M, Mitchell N, Chryssouli Z, Giamarellou H (2010) Presence of plasmid-mediated quinolone resistance in Klebsiella pneumoniae and Escherichia coli isolates possessing blaVIM-1 in Greece. Int J Antimicrob Agents 36: 252-254.

4. Pan JC, Ye R, Meng DM, Zhang W, Wang HQ, et al. (2006) Molecular characteristics of class 1 and class 2 integrons and their relationships to antibiotic resistance in clinical isolates of Shigella sonnei and Shigella flexneri. J Antimicrob Chemother 58: 288-296.

5. Hu LF, Li JB, Ye Y, Li X (2007) Mutations in the GyrA subunit of DNA gyrase and the ParC subunit of topoisomerase IV in clinical strains of fluoroquinolone-resistant Shigella in Anhui, China. J Microbiol 45: 168-170.

6. Robicsek A, Strahilevitz J, Sahm DF, Jacoby GA, Hooper DC (2006) qnr prevalence in ceftazidime-resistant Enterobacteriaceae isolates from the United States. Antimicrob Agents Chemother 50: 2872-2874.

7. Pu XY, Pan JC, Wang HQ, Zhang W, Huang ZC, et al. (2009) Characterization of fluoroquinolone-resistant Shigella flexneri in Hangzhou area of China. J Antimicrob Chemother 63: 917-920.
