# Supplementary material for: Dominant serotype distribution and antimicrobial resistance profile of Shigella spp. in Xinjiang, China
Source: PLoS One. 2018 Apr 3;13(4):e0195259. doi: 10.1371/journal.pone.0195259 (PMC5882154; doi:10.1371/journal.pone.0195259)
Supplement: S2 Table — (DOCX) [file pone.0195259.s003.docx]

**S2 Table. MDR classes of *Shigella* strains isolated in Xinjiang, China.**

| Antimicrobial classes* | Antimicrobial classes quantity | Antimicrobial resistance No. | |
| --- | --- | --- | --- |
|  |  | *S. flexneri* | *S. sonnei* |
| AMP/AMI/PEN | 3 | 1 | 3 |
| AMP/CEP/PEN | 3 | 3 | 0 |
| AMP/FPI/PEN | 3 | 1 | 1 |
| AMP/PEN/QUI | 3 | 1 | 0 |
| AMP/PEN/TET | 3 | 111 | 0 |
| CEP/MON/PEN | 3 | 0 | 2 |
| CEP/PEN/TET | 3 | 1 | 0 |
| FPI/PEN/TET | 3 | 9 | 0 |
| FPI/PEN/TET | 3 | 1 | 0 |
| FPI/QUI/TET | 3 | 2 | 0 |
| AMI/FPI/PEN/TET | 4 | 2 | 37 |
| AMI/FPI/QUI/PEN | 4 | 2 | 0 |
| AMP/FPI/PEN/TET | 4 | 86 | 0 |
| AMP/FPI/QUI/TET | 4 | 1 | 0 |
| AMP/MON/PEN/PEN | 4 | 1 | 0 |
| AMP/CEP/PEN/QUI | 4 | 1 | 0 |
| AMP/CEP/PEN/TET | 4 | 10 | 0 |
| AMP/PEN/QUI/TET | 4 | 32 | 0 |
| FPI/PEN/PEN/TET | 4 | 2 | 2 |
| AMI/AMP/CEP/PEN/TET | 5 | 3 | 0 |
| AMI/AMP/FPI/PEN/TET | 5 | 1 | 1 |
| AMP/CEP/FPI/MON/PEP | 5 | 1 | 0 |
| AMP/CEP/FPI/PEN/TET | 5 | 3 | 0 |
| AMP/CEP/PEN/QUI/TET | 5 | 2 | 0 |
| AMP/FPI/PEN/QUI/TET | 5 | 28 | 0 |
| APM/CEP/MON/PEN/TET | 5 | 3 | 0 |
| CEP/FPI/MON/PEN/TET | 5 | 0 | 13 |
| AMI/AMP/CEP/FPI/PEN/TET | 6 | 4 | 0 |
| AMI/CEP/FPI/MON/PEN/TET | 6 | 1 | 0 |
| AMI/CEP/FPI/PEN/QUI/TET | 6 | 2 | 0 |
| AMP/CEP/FPI/MON/PEP/TET | 6 | 2 | 0 |
| AMP/CEP/FPI/PEN/QUI/TET | 6 | 5 | 0 |
| AMP/CEP/MON/PEN/QUI/TET | 6 | 1 | 0 |
| AMI/CEP/FPI/MON/PEN/QUI/TET | 7 | 1 | 0 |
| AMP/CEP/FPI/MON/PEN/QUI/TET | 7 | 6 | 0 |
| Total |  | 330 | 59 |

* AMI, aminoglycosides; AMP, amphenicols; CEP, cephems; MON, monobactams; PEN, penicillins; TET, tetracyclines; QUI, fluoroquinolones.
